# High-Fat Diet Induces Unexpected Fatal Uterine Infections in Mice with aP2-Cre-mediated Deletion of Estrogen Receptor Alpha

**DOI:** 10.1038/srep43269

**Published:** 2017-02-24

**Authors:** Zsofia Ban, Paul Maurischat, Verena Benz, Sarah Brix, Anna Sonnenburg, Gerhard Schuler, Robert Klopfleisch, Michael Rothe, Jan-Åke Gustafsson, Anna Foryst-Ludwig, Ulrich Kintscher

**Affiliations:** 1 Charité-Universitaetsmedizin Berlin, Institute of Pharmacology, Center for Cardiovascular Research, Berlin, Germany; 2 Charité-Universitaetsmedizin Berlin, Institute of Clinical Pharmacology and Toxicology, Berlin, Germany; 3 Clinic for Obstetrics, Gynaecology and Andrology of Large and Small Animals, Faculty of Veterinary Medicine, Justus-Liebig-Universität Gießen, Germany; 4 Department of Veterinary Pathology, College of Veterinary Medicine, Freie Universität Berlin, Berlin, Germany; 5 Lipidomix GmbH, Berlin, Germany; 6 University of Houston, Center for Nuclear Receptors and Cell Signaling, Houston, TX, USA; 7 DZHK (German Centre for Cardiovascular Research), partner site Berlin, Germany

## Abstract

Estrogen receptor alpha (ERα) is a major regulator of metabolic processes in obesity. In this study we aimed to define the relevance of adipose tissue ERα during high-fat diet (HFD)-induced obesity using female aP2-Cre^−/+^/ERα^fl/fl^ mice (atERαKO). HFD did not affect body weight or glucose metabolism in atERαKO- compared to control mice. Surprisingly, HFD feeding markedly increased mortality in atERαKO mice associated with a destructive bacterial infection of the uterus driven by commensal microbes, an alteration likely explaining the absence of a metabolic phenotype in HFD-fed atERαKO mice. In order to identify a mechanism of the exaggerated uterine infection in HFD-fed atERαKO mice, a marked reduction of uterine M2-macrophages was detected, a cell type relevant for anti-microbial defence. In parallel, atERαKO mice exhibited elevated circulating estradiol (E2) acting on E2-responsive tissue/cells such as macrophages. Accompanying cell culture experiments showed that despite E2 co-administration stearic acid (C18:0), a fatty acid elevated in plasma from HFD-fed atERαKO mice, blocks M2-polarization, a process known to be enhanced by E2. In this study we demonstrate an unexpected phenotype in HFD-fed atERαKO involving severe uterine bacterial infections likely resulting from a previously unknown negative interference between dietary FAs and ERα-signaling during anti-microbial defence.

There is a growing body of evidence from human and rodent studies for a crucial role of estrogen and estrogen receptors (ERs), in particular ERα, in the regulation of body weight^1^. Menopause is associated with loss of ligand-mediated ER-signaling causing increased adiposity and body fat redistribution^2^. The reconstitution of regular ER-signaling by hormone replacement can prevent menopause-mediated weight gain, and results in fat redistribution to subcutaneous fat depots, and improvement of insulin sensitivity^3^. According to these studies, female rodents become obese after undergoing ovariectomy, and replacement of estrogens abrogates BW-gain^4^. Similar to the ligand-deficient models, the ERα-knock out mice exhibit increased BW and fat mass without a concomitant change in food consumption but a significantly reduction of energy expenditure compared to wild-type animals^5^. Deficiency of ERβ also results in increased body weight^6^. In contrast to ERα-knock out mice, loss of ERβ leads to an improvement of insulin and glucose metabolism^6^. Despite the metabolic characterization of both isoforms, it has become increasingly clear that ERα is the predominant regulator of body weight and glucose/lipid metabolism^7^.

ERα mediates its metabolic actions via the central nervous system (CNS) and via peripheral organs/cells such as adipose tissue and macrophages^7^,^8^,^9^. Regarding ERα’s CNS actions, Xu and colleagues previously phenotyped CNS-specific ERα knock-out mice^10^. The authors demonstrated that floxed-ERα mice crossed with Nestin-Cre transgenic mice, show ERα loss in most brain regions, exhibit decreased locomotor activity, abdominal obesity and reduced energy expenditure, a phenotype similar to complete ERα-deficient animals^10^. ERα-deletion in neurons of the ventromedial hypothalamic nucleus (VMH) resulted in reduced energy expenditure, and deletion in pro-opiomelanocortin (POMC) neurons led to hyperphagia^10^. These data delineate the metabolic CNS-effects of ERα involving an increase of energy expenditure and a suppression of food intake. The peripheral metabolic actions of ERα are less well understood. Ribas and colleagues demonstrated that a loss of ERα in myleoid cells results in increased adipose tissue mass, insulin resistance and glucose intolerance^9^. In addition, ERα acts in white adipose tissue, and enhances subcutaneous white adipose tissue distribution while decreasing overall adipose mass involving a reduced FFA-uptake, lipid synthesis and increasing lipolysis^7^,^8^,^11^. In addition, ERα protects against adipose tissue inflammation and fibrosis^8^.

To determine the role of adipose tissue ERα for body weight regulation and whole body insulin and glucose metabolism, mice were generated lacking ERα in adipose tissue (aP2- Cre^−/+^/ERα^fl/fl^ mice) and control littermates (aP2-Cre^−/−^/ERα^fl/fl^ mice) (wt). These mice were kept on a high fat diet (HFD). Surprisingly, atERαKO mice on HFD did not differ in body weight, insulin sensitivity or glucose tolerance compared to wt-mice. More importantly, HFD feeding markedly increased mortality in atERαKO compared to wt controls and atERαKO mice fed control diet (CD). HFD-induced mortality resulted from massive and fatal bacterial uterine infections in atERαKO mice. We identified that dietary fatty acids markedly attenuate ERα-signaling in macrophages accompanied with impaired neutrophil clearance during bacterial infection subsequently leading in a multifactorial context to aggravated infections.

In summary, this study identifies an unexpected phenotype in HFD-fed atERαKO mice pointing towards a crucial interaction between dietary fatty acids and ERα-signaling during bacterial infections.

## Results

### No metabolic phenotype but increased mortality in HFD-fed atERαKO mice

**Figure 1:**
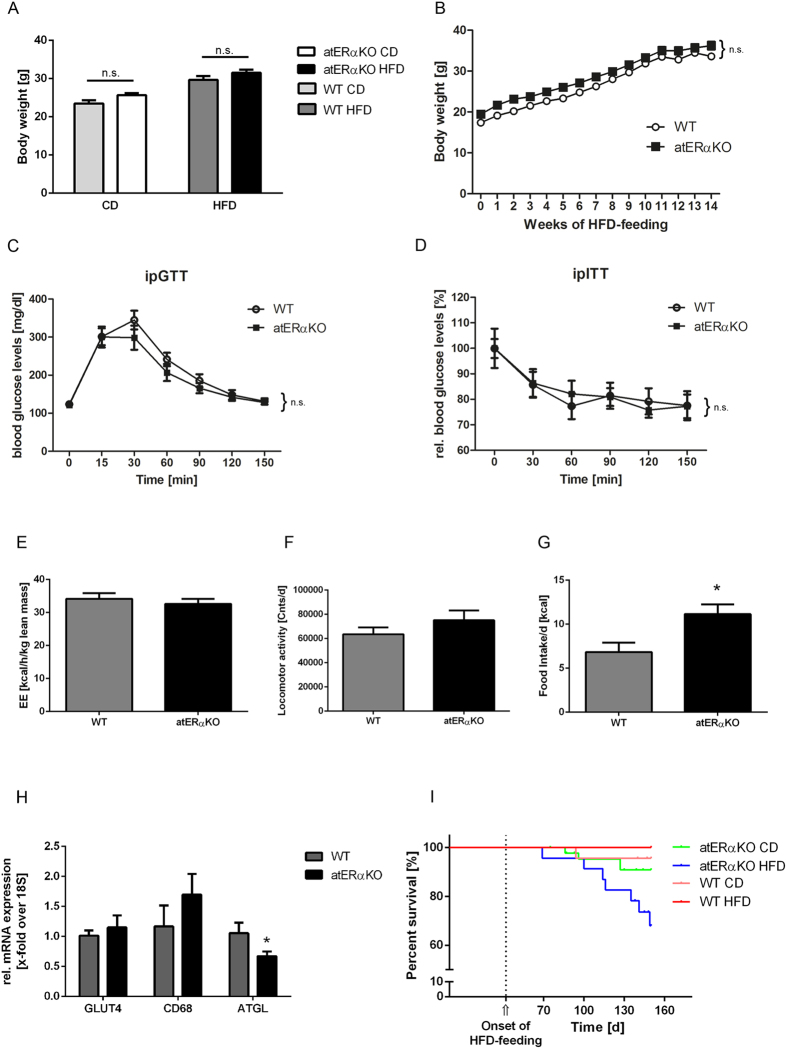
Metabolic phenotyping of female atERαKO mice. (**A**) Body-weight of atERαKO- compared to wt-mice fed a control (CD) or a high-fat diet (HFD) at the age of 15 weeks. (n = 5 (CD) and 17–19 (HFD)). **(B)** Body weight development during HFD-feeding, showing no differences between genotypes. **(C,D)** Intraperitoneal glucose- **(C)** and insulin- **(D)** tolerance test (n = 9–10). No differences occurred in energy expenditure **(E)** and in locomotor activity **(F)** between the genotypes, while atERαKO mice showed an increase in food intake **(G)** (n = 10). **(H)** Gene expression analyses in white adipose tissue of wt and atERαKO mice (n = 5–8). **(I)** Survival curves of atERαKO- and wt-mice on CD and HFD. Onset of HFD-feeding at 42 days of age is indicated in the graph. n.s. = non-significant (P > 0.05, 2-way-ANOVA (Bonferroni-posttest) and 2-way-ANOVA with repeated measures (Bonferroni-posttest)). ^*^P < 0.05 vs. wt (two-tailed t-test).

Metabolic baseline characterization of 6 weeks old female atERαKO mice resulted in the expected metabolic phenotype with increased body weight (BW) and decreased energy expenditure (Table 1). However, in 15 weeks old wt- and atERαKO mice on control diet (CD) BW-differences disappeared (Fig. 1A). More importantly, HFD-induced increase of BW after 14 weeks feeding did not differ between wt- and atERαKO mice (Fig. 1A,B). Along this line, no differences could be detected for glucose tolerance and insulin sensitivity between wt- and atERαKO mice after HFD-feeding (Fig. 1C,D). Neither energy expenditure nor locomotor activity, assessed by metabolic cage experiments, showed any alteration in the HFD-fed atERαKO group when compared to control (Fig. 1E,F). However, mutant mice displayed an increase in food intake (Fig. 1G). These data appeared highly controversial to recently published results about the role of ERα in adipose tissue pointing towards a protective action of ERα against BW gain and HFD-mediated glucose intolerance^7^. Expression analysis of metabolically relevant genes in white adipose tissue showed only modest effects of ERα, reaching statistical significance only for the reduction of ATGL expression in the absence of ERα (Fig. 1H). These data corroborate our previous findings pointing to a pivotal role of ERα in lipolysis^12^. Surprisingly, after the onset of HFD-feeding we observed a markedly increased mortality in HFD-fed atERαKO mice compared to atERαKO mice on CD and to wt-mice (Fig. 1I). Together these data suggest that HFD-feeding induces a fatal pathology in atERαKO which likely impacts on physiological metabolic processes.

### HFD mediates fatal uterine infections in atERαKO mice

**Figure 2:**
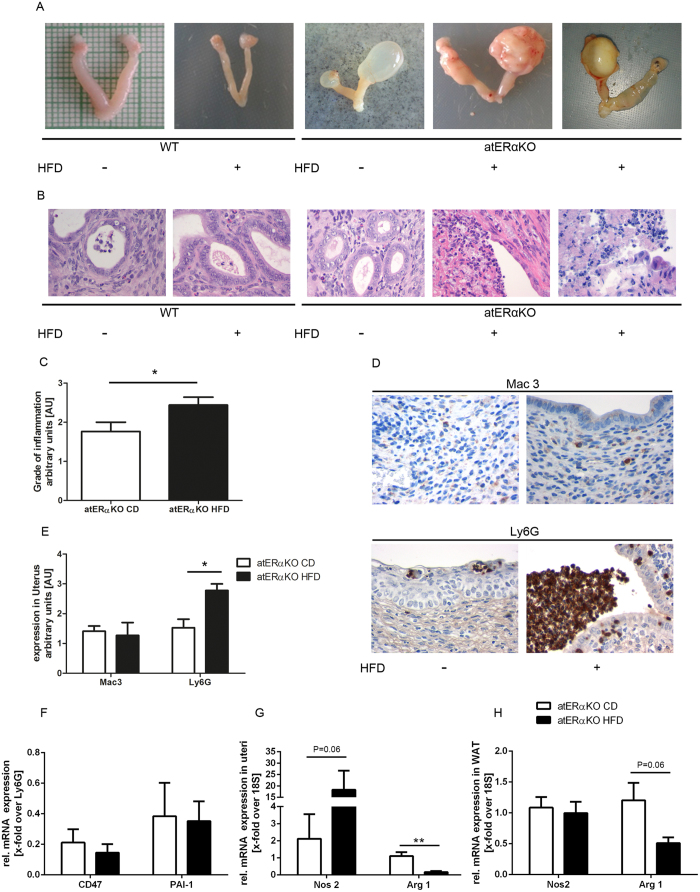
Characterization of uterine phenotype. **(A,B)** Representative macro- and microscopic images of uteri of atERαKO vs. wt-mice fed a CD or HFD. **(C)** Quantification of the grade of inflammation (n = 9–17). Legend: 0 = no inflammation; 1 = beginning inflammation; 2 = visible inflammation; 3 = severe inflammation **(D)** Representative Mac3- and Ly6G-stainings. **(E)** Quantification of macrophages (Mac3) and neutrophils (Ly6G) in uteri of atERαKO mice (n = 9–17). Legend: 0 = no immune cells; 1 = few immune cells; 2 = high quantity of immune cells; 3 = very high quantity of immune cells **(F)** Relative mRNA-expression of CD47 and PAI-1 normalized to Ly6G mRNA-expression in uteri, comparing atERαKO mice on CD or HFD (n = 4–7). Relative Nos2 and Arg1 mRNA expression in uteri **(G)** and white adipose tissue **(H)** of atERαKO mice (n = 7–8). ^*^P < 0.05, ^**^P < 0.01 two-tailed t-test or 2-way-ANOVA (Bonferroni-posttest).

Autopsies of wt- and atERαKO mice revealed massive uterine fluid accumulation in CD-fed atERαKO mice compared to wt-mice as previously described (Fig. 2A)^13^. More importantly, HFD-feeding aggravated these uterine processes in atERαKO mice leading to a destructive, pus-filled swelling of the uterus and uterine appendages in line with a severe bacterial infection in all HFD-fed atERαKO mice (Fig. 2A, right panels). In consonance, microbiological analysis of the uterine fluid of HFD-fed atERαKO mice showed the presence of commensal microbes including Enterococcus sp. and bacterial DNA for E.coli (data not shown). These results were confirmed by histological analysis (Fig. 2B) demonstrating massive cellular infiltration of the uterine wall in HFD-fed atERαKO accompanied by destruction of the intramural glandular and epithelial structure (Fig. 2B, right panels). The grade of uterine inflammation as determined by microscopic analysis (for detailed protocol see methods) was significantly higher in atERαKO mice fed HFD vs. CD (Fig. 2C). CD- or HFD-fed wt-mice showed no evidence for uterine inflammation (data not shown). Bacterial uterine infections in HFD-fed atERαKO mice were characterized by pronounced neutrophil accumulation associated with a low number of macrophages in the uterine wall (Fig. 2D,E). Neutrophils initially recruited to the site of bacterial infection are usually cleared by macrophages to initiate physiological resolution and to prevent exaggeration of inflammation, a process called efferocytosis^14^. Regular macrophage-mediated neutrophil clearance depends on specific neutrophil marks to engage particularly M2-macrophages^14^,^15^. To understand the neutrophil-macrophage interaction resulting in prominent uterine neutrophil accumulation in HFD-fed atERαKO mice in our study, the regulation of neutrophil signals in uteri was first studied. So called “Don’t eat me” signals” on viable neutrophils such as CD47 and PAI-1^14^ did not differ in uteri from CD- and HFD-fed atERαKO mice (Fig. 2F). However, mRNA expression analysis of M1- and M2 macrophage markers in uteri from atERαKO mice revealed a statistical non-significant increase of the M1-marker Nos2, but more importantly a highly significant reduction of the M2-marker Arg1 by HFD (Fig. 2G). These data suggest that HFD does not directly affect signals on neutrophils but more likely augments neutrophil presence at the site of infection by an indirect shift in macrophage polarization towards pro-inflammatory conditions and resulting in a significant repression of M2 macrophages. A similar trend could be registered in the expression of Arg1 in white adipose tissue, however, no alteration of Nos2 expression could be detected (Fig. 2H). These data suggest that the macrophage phenotype may be regulated in a tissue-specific manner in our model, and that the bacterial environment is, at least in part, required for the enhanced presence of M1 macrophages in the uteri.

To sum up, we show that HFD-feeding promotes a severe bacterial uterine infection in atERαKO mice likely causing higher mortality. Uterine bacterial infection in HFD-fed atERαKO mice is characterized by an excess of uterine neutrophil and M1/M2-macrophage imbalance.

### HFD-feeding impairs amplified ERα signaling in atERαKO mice

**Figure 3:**
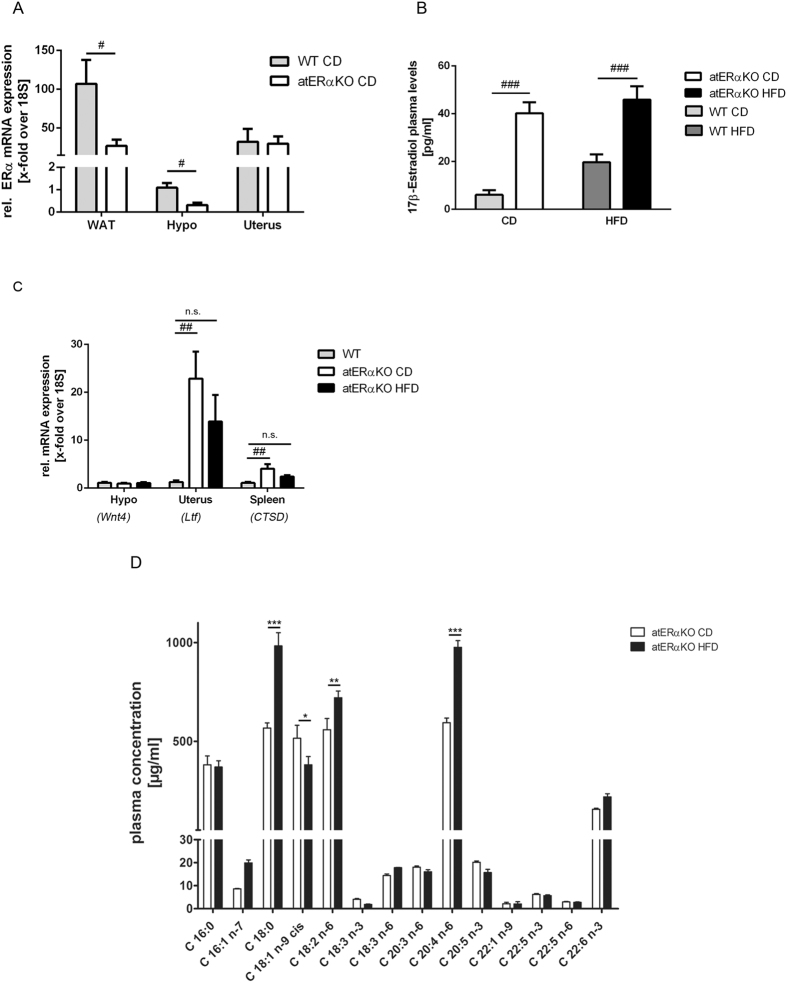
ERα-signaling and lipid profile of atERαKO mice. **(A**) Relative ERα mRNA-expression in indicated tissues (n = 5). **(B)** 17β-estradiol levels in plasma of female atERαKO- and wt-mice (n = 5–10). **(C)** Relative mRNA expression of ERα-target genes in hypothalamus (*Wnt4*), uterus (*Ltf*) and spleen (*CTSD*) in CD or HFD-fed atERαKO- and wt-mice (WT) (n = 4–8). **(D)** Measurement of plasma concentration of fatty acids in HFD-fed atERαKO mice vs. CD-fed (n = 3). ^*^P < 0.05, ^**^P < 0.01, ^***^P < 0.001 vs. atERαKO CD; ^#^P < 0.05, ^###^P < 0.001 vs. WT; one-way ANOVA, 2-way-ANOVA or unpaired t-test.

In order to understand the underlying mechanism leading to fatal bacterial infections in atERαKO mice through “simple” HFD-feeding, additional plasma and expression analysis in wt- and atERαKO mice were performed. Antonson and colleagues recently found that aP2-Cre-mediated deletion of ERα not only results in ERα depletion in adipose tissue but also in the hypothalamus^13^. In agreement with these findings, ERα mRNA expression was significantly reduced in white adipose tissue and hypothalamus but not in the uterus (Fig. 3A). According to the study of Antonson and colleagues, we found that atERαKO female mice are infertile and have no proper estrous cycle compared to wt, as demonstrated by evaluation of vaginal smear (Fig. S1). E2 levels were also significantly higher in atERαKO- compared to wt-mice (CD: 40.2 ± 4.6 vs. 6.1 ± 2.0 pg/ml; HFD: 45.9 ± 5.7 vs. 19.7 ± 3.4 pg/ml; P < 0.001) suggesting enhanced estrogen signaling in atERαKO mice on CD and HFD (Fig. 3B). Accordingly, E2/ERα-target genes were markedly upregulated in CD-fed atERαKO mice, in organs still expressing ERα such as uterus and spleen (Fig. 3C, white bars). Of note was that under HFD ERα-target gene induction in uterus and spleen did no longer reach statistical significance (Fig. 3C, black bars) despite high E2-levels implicating impairment of ERα- signaling by HFD. To identify potential mediators in HFD responsible for disturbed ERα-signaling plasma lipid analysis using HPLC/triplequad mass spectrometry was performed. As shown in Fig. 3D, HFD feeding resulted in the regulation of distinct plasma fatty acids (FA) among which C18:0 (stearic acid), C18:2n6 (linoleic acid), and C20:4n6 (arachidonic acid) were significantly upregulated, and only C18:1n9 (oleic acid) was downregulated. Taken together, atERαKO mice demonstrated reduced hypothalamic ERα expression levels associated with increased circulating E2-levels leading to enhanced ERα signaling in ERα expressing tissue. HFD-feeding induced a distinct plasma FA-profile accompanied by a blockade of ERα signaling.

### C18:0 (stearic acid) impairs E2-mediated macrophage polarization and phagocytotic activity

**Figure 4:**
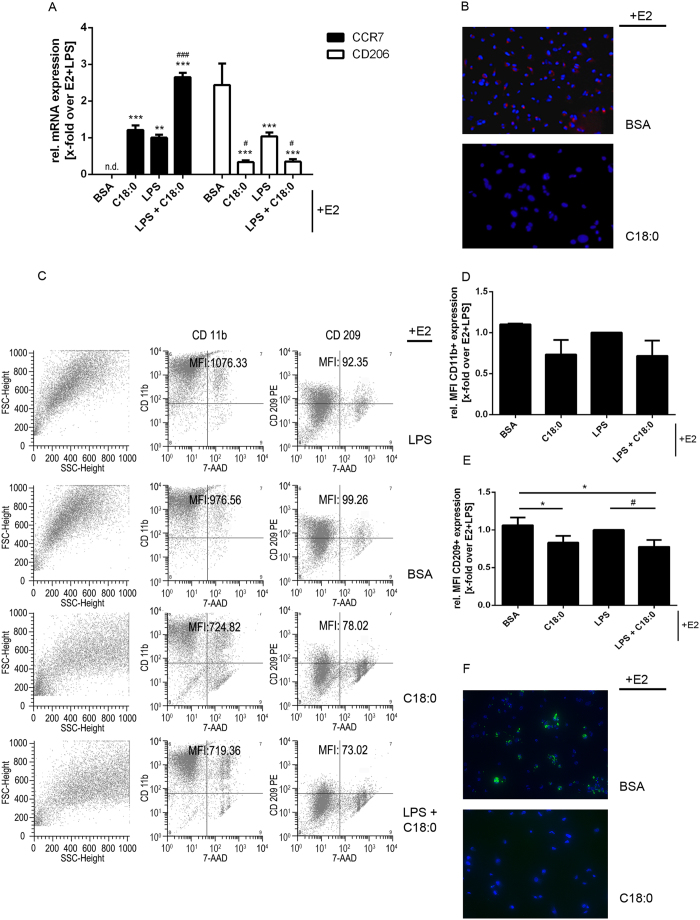
Impact of stearic acid on macrophage polarization (and function). Characterization of M1- and M2-markers on differentiated THP-1 cells after stimulation with estradiol (E2)+BSA, E2+C18:0, E2+LPS and E2+LPS+C18:0. **(A)** Relative mRNA-expression of CCR7 (M1) and CD206 (M2) over E2+LPS stimulated cells. **(B)** Representative images of CD209 (M2-marker) staining of E2+BSA or E2+C18:0 stimulated THP-1 cells. **(C)** Representative FACS-dot plots of CD11b and CD209-staining. First column represents unstained cells in forward (FSC) and side scattered blots (SSC); second column shows staining of CD11b- (y-axis) and 7AAD- (x-axis) staining. The third column is analogous to the second one for CD209-staining. Average MFI of viable (7AAD negative) CD11b positive cells (left upper quarter of the blots) and MFI of viable CD209 cells are indicated. **(D)** Relative mean-fluorescence-intensity of cd11-positive cells and **(E)** relative mean-fluorescence-intensity of cd209-positive cells (x-fold induction over E2/LPS-stimulated cells). **(F)** Representative images of phagocytotic analysis of E2+BSA or E2+C18:0 stimulated THP-1 cells. ^**^P < 0.01, ^***^P < 0.001 vs. E2+BSA; ^#^P < 0.05, ^###^P < 0.001 vs. E2+LPS (n = 3; one-way-ANOVA).

C18:0 (stearic acid) induces pro-inflammatory processes in macrophages^16^ whereas E2 exerts anti-inflammatory actions in these cells^17^. Plasma level of C18:0 showed the strongest up-regulation by HFD feeding in our model. To answer whether the HFD-induced rise in plasma FAs accounts for M1/M2-macrophage imbalance, disturbed neutrophil depletion and exaggerated bacterial inflammation in atERαKO mice, the effect of C18:0 on macrophage M1/M2 polarization in the presence of high E2 was characterized. As previously mentioned, depletion of CD206-positive macrophages (alternatively activated/M2 polarized) can lead to aggravation of inflammatory processes characterized by accumulation of neutrophils at the inflammatory sites. Stimulation of THP-1 macrophages with C18:0 on an E2-background significantly induced CCR7 mRNA levels, a marker for M1 macrophages, and significantly suppressed the expression of CD206, independently from the presence of lipopolysaccharide (LPS) (Fig. 4A). Immunostaining (Fig. 4B) and flow cytometry (FACS) - analysis (Fig. 4C,E) of CD209 (M2-marker^18^,^19^) in THP-1 cells, confirmed the repression of M2-polarization by C18:0. FACS analyses show that the MFI (mean fluorescence intensity) of viable (7AAD negative) CD209 positive cells (left upper quarter of the third column of plots) is significantly decreased from 92.35 to 73.02 (E2 and LPS co-treatment) and from 99.26 to 78.02 (only E2 co-treatment) due to C18:0 action. CD11b, an activation marker and an important integrin for immune cell adhesion, was not significantly regulated (Fig. 4C,D), but shows a slight tendency towards a decrease of CD11b induced by C18:0. Stimulation with the FA leads to a decrease of the average MFI from 1076.33 to 719.36 (E2 and LPS co-treatment) and from 976.56 to 724.82 (only E2 co-treatment). These data show that C18:0 is capable of blocking E2-mediated M2-macrophage polarization, a process important for E2’s anti-inflammatory capacity. Furthermore the functionality of macrophages in the presence of C18:0 was tested. The addition of the fatty acid negatively influenced phagocytotic activity of THP-1 macrophages (Fig. 4F). Similar effects on the functionality of macrophages were observed in mice lacking ERα in myeloid cells^9^.

### C18:0 induces acylation of ERα-immunoprecipitates and inhibits ERE-activity

**Figure 5:**
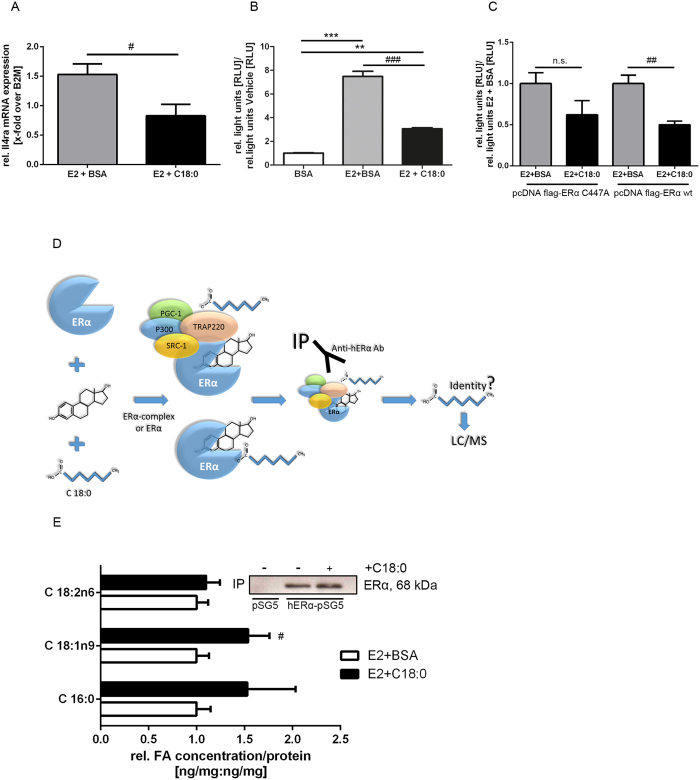
Stearic acid modulation of ERα transcriptional activity. **(A)** Relative mRNA expression of IL4-receptor after stimulation with E2 and C18:0 in primary murine bone marrow-derived macrophages (n = 7–9 per condition). **(B)** Inhibition of ligand-dependent activation of ERE by C18:0 in THP-1 cells (n = 3–8 per condition). **(C)** Inhibition of E2-induced ERE-activity by C18:0 in cells transiently expressing mutated C447A (left bars) or wt (right bars) ERα. **(D)** Experimental settings of HPLC/MS lipid analysis from protein (ERα) precipitates **(E)** Relative concentration of main fatty acids bound to ERα per 1 mg protein. (inlay: IP- and transfection-controls by Western-Blot (cropped) of transfected HeLa cells with pSG5 and hERα-pSG5 plasmids; full-length blot was provided as supplemental data). ^#^P < 0.05, ^##^P < 0.01, ^###^P < 0.001 vs. E2+BSA; ^**^P < 0.01, ^***^P < 0.001 vs. vehicle; (one-way-ANOVA or unpaired t-test).

How does C18:0 impair ERα signaling and E2-mediated macrophage polarization? The Th2 cytokine interleukin 4 (IL4) and the corresponding IL4-receptor promote M2-macrophage polarization^20^. E2-dependent IL4-receptor mRNA expression in primary murine bone marrow-derived macrophages was significantly reduced by co-stimulation with C18:0 (Fig. 5A). In accordance, C18:0 potently blocked E2-dependent ERE-activity in ERα-overexpressing THP-1 macrophages (Fig. 5B). Acylation of ERα has been recently shown to directly regulate ERα-dependent transcriptional activity via the ERα cysteine (C) residue 447^21^. Mutation of ERα C447 (C447A) resulted in a noticeable attenuation of C18:0’s inhibitory action on E2-dependent ERE-activity in comparison to ERα wt (Fig. 5C) suggesting that this residue is, at least partly, involved in C18:0’s repressive actions on ERα. To see whether C18:0 impairs ERα-activity via binding to the receptor complex, we performed ERα-immunoprecipitation (IP) experiments after E2 ± C18:0 stimulation followed by HPLC/triplequad mass spectrometry for FA-detection (Fig. 5D,E). Co-treatment with C18:0 did result in an increased content of a FA in the protein precipitate suggesting an enhanced interaction between FAs and the receptor complex. Interestingly, binding of the monounsaturated FA C18:1n9 to ERα was significantly induced by C18:0 stimulation whereas C18:0 itself was not detectable in IPs suggesting FA-desaturation after stimulation. These results indicate, C18:0 potently inhibits E2-dependent ERα-transcriptional activity potentially involving acylation of the receptor or the receptor multi-protein complex.

## Discussion

ERα exerts multiple metabolic actions in adipose tissue^7^. To prove that adipose ERα plays a crucial role for whole body metabolism, we investigated the role of ERα in a tissue specific knock-out model using aP2-Cre^−/+^/ERα^fl/fl^ mice. A similar approach has previously been conducted by Davis and colleagues. This demonstrated that, in adiponectin promoter driven-Cre transgenic mice crossed with floxed ERα mice, a lack of ERα in adipose tissue results in an increase of gonadal adipose tissue mass, enhanced adipose tissue fibrosis and inflammation, and in glucose intolerance in male mice^8^. Due to an unexpected mortality increase in HFD-fed atERαKO mice, based on a severe bacterial infection, interpretation of the metabolic data in our study are limited which prompted us to focus on the etiology of the uterine infection in our model. The observed discrepancies between Davis and our data likely result from differences in the utilized Cre lines.

In line with Mullican and colleagues and other investigators, we observed that the aP2-Cre model lacks specificity for adipose tissue, reducing ERα mRNA levels also in the hypothalamus^22^. Hypothalamic ERα appears to be important for regular uterine development. Antonson and colleagues recently showed that female aP2-Cre^−/+^/ERα^fl/fl^ fed a CD display a reproductive phenotype involving infertility, hyperplasia and hydrometra of the uterus^13^. Moreover, other knock-out models of ERα in the brain such as the CamKIIa-Cre-mediated model, also display an enlarged and fluid-filled uterus^23^. Uterine pathologies observed in these models, including this, presumably involve a lack of a central ERα-dependent negative feedback resulting in elevated E2-levels subsequently affecting uterine development. Furthermore, elevated E2-levels likely conceal some of the metabolic effects usually expected in mice lacking ERα exclusively in adipose tissue.

The present study, however, is the first report of increased mortality in aP2-Cre^−/+^/ERα^fl/fl^ mice. In contrast to previous studies, a severe bacterial, E. coli/Enterococcus sp. positive, uterine infection was observed. Since HFD-feeding is the obvious difference between Antonson’s^13^ and this study, analysis was conducted to see whether HFD content, in particular FAs, act as pathogenic mediators of disturbed bacterial defense. HFD is well known to induce pro-inflammatory processes on the tissue- and systems level^24^,^25^. Indeed, in contrast to atERαKO mice on CD, a severe inflammatory response was identified in uterine tissue from mice fed a HFD characterized by massive neutrophil accumulation and a reduced number of uterine M2-macrophages. Defects of neutrophil clearance have been linked to severe inflammation including sepsis^26^. Neutrophil clearance from the infectious site is a complex process predominantly orchestrated by neutrophils themselves and macrophages^14^,^26^,^27^. Anti-inflammatory (M2 polarized) macrophages seem to especially be required for neutrophil clearance and play a crucial role in the resolution of inflammation^15^,^28^. It was hypothesized that the HFD-induced atERαKO mice phenotype is mainly a result of HFD-mediated blockade of anti-inflammatory E2-ERα-actions. E2-dependent ERα activation usually induces M2-macrophage polarization, and thus attenuating inflammatory responses^9^,^17^. Under a normal diet, elevated E2 levels in atERαKO mice lead to enhanced E2-signaling and likely to containment of the inflammatory response. In contrast under HFD, it was shown that C18:0, most strongly up-regulated in plasma from HFD-fed mice, inhibits E2-induced ERα-activation significantly blocking E2-stimulated M2-macrophage polarization, and probably exaggerating inflammation. In consonance, C18:0 has been already described to possess pro-inflammatory effects on macrophages^16^. In summary these data suggest that the inflammatory control by E2 is abolished by C18:0 triggering the multifactorial process that induces fatal bacterial infections in this model. Depending on the used ap2-Cre strain, it is known that the targeted gene can be also affected in macrophages^29^,^30^ opening the possibility of reduced ERα expression. However, this seems not to be the case in the aP2-Cre strain used in our study. Using the identical strain, Mayoral *et al*. recently showed that macrophages in this strain do not express Cre-recombinase^31^. In line with this findings, in CD-fed aP2-Cre^−/+^/ERα^fl/fl^ mice we still observed a robust induction of the ERα target gene CTSD by E2 in the spleen, a gene highly expressed in macrophages. These data suggest an existing E2 response in macrophages in CD-fed aP2-Cre^−/+^/ERα^fl/fl^ mice rendering it unlikely that macrophages lack ERα.

Finally, the molecular mechanism of C18:0’s repressive action on E2-ERα-signaling was investigated. Stearoylation of proteins has been recently identified as a new determinant of protein function^32^. Senyilmaz and colleagues demonstrated that C18:0 stearoylates the human transferrin receptor 1 (TFR1), a protein important for mitochondrial function^32^. Other reports had previously shown the existence of an interaction between ERα and long-chain fatty acids^33^. Stearic acid was described to inhibit E2-effects by downregulation of ERα in hypothalamic tissue^33^, while palmitic acid (C16:0) promotes extra-nuclear action of ERα and prevents its degradation through acylation^21^. In the work of La Rosa and colleagues, the lack of ERα-palmitoylation at cysteine (C) residue 447 was shown to repress its transcriptional activity in HEK293 cells^21^. Contrastingly, we observed a significant decrease in E2-induced ERE-activity with C18:0 in differentiated THP-1 macrophages, also likely mediated via C447. HPLC/triplequad MS-based profiling of FAs bound to immunoprecipitated ERα demonstrated a significant protein-FA interaction between ERα, its protein co-factors and C18:1n9 (oleic acid), the main metabolite of C18:0 after enzymatic transformation through the stearoyl-CoA desaturase-1 (SCD-1)^34^. Predominance of cellular C18:1n9 after C18:0 stimulation is not surprising, since it has long been known that desaturation of C18:0 is greater than compared to other saturated FAs^35^. Up until now, the following mechanism can be proposed: challenge of macrophages with C18:0 leads to repression of ERα-transcriptional activity potentially involving the direct interaction between FAs and the of ERα transcriptional protein complex. The relative contribution of the direct FA – ERα-complex interaction to the observed phenotype in HFD-fed atERαKO mice requires further experiments.

The importance of these data is further supported by clinical observations in female dogs. In sexually mature female dogs, pyometra is a common disease entity^36^. Up until now, the pathogenesis has been linked to increased estradiol levels and prolonged progesterone stimulation^36^. In light of our findings, it might be worth to investigate the relevance of dietary FAs in conjunction with high E2/progesterone levels for the pathogenesis of canine pyometra. Along this line, these findings might also be relevant for bacterial uterine infections in humans when conditions occur in which frequent HFD consumption is combined with high plasma E2-levels, e.g. during pharmacological application.

This study identifies an unexpected phenotype of atERαKO mice fed HFD characterized by increased mortality likely due to fatal bacterial uterine infections driven by commensal microbes. This phenotype points towards a previously unknown interaction of ERα signaling and FAs in macrophages. In particular, it was shown that C18:0 provided by HFD impairs E2-ERα action in macrophages with a concomitant perturbation of M2-macrophage polarization usually required for regular neutrophil-mediated bacterial defense. In accompanying *in-vitro* experiments we identified a direct interaction between FAs and the intracellular ERα transcriptional complex associated with a C18:0-mediated inhibition of ERα transcriptional activity and E2-dependent regulation of genes involved in M2 macrophage polarization. These data suggests a new mechanism of how FAs are capable of disturbing macrophage E2-ERα-signaling thereby aggravating commensal bacterial infections at predilection sites.

Therefore, it is suggested a new mechanism of dietary FA interference with ERα resulting in the impairment of E2-mediated anti-inflammatory actions and the promotion of severe bacterial uterine infections.

## Materials and Methods

### Mice

Female adipose tissue-specific (aP2) - mice were generated by crossing B6.Cg-Tg(Fabp4-cre)1Rev/J mice with B6.129X1-Esr1tm1Gust (ERα^fl/fl^) mice, kindly provided by J.-A. Gustafsson (University of Houston, TX, USA). ERα^fl/fl^/aP2-Cre^−/+^ (atERαKO) and control littermates ERα^fl/fl^/aP2-Cre^−/−^ (wt) were used for all experiments. Animals were maintained in a temperature-controlled facility with a 12 h dark/light cycle. At the age of 6 weeks, a group of atERαKO and wt-mice (n = 24), were challenged for 14 weeks with a HFD (60% kcal from fat, Brogaarden, Lynge, Denmark). The HFD groups and the CD were both fed ad-libitum. Metabolic phenotyping was performed with a metabolic cage system (TSE- Systems GmbH, Bad Homburg, Germany), based on indirect calorimetry as described before^12^. Body composition was assessed by nuclear magnetic resonance imaging (Echo MRI mouse, Echo Medical Systems, Houston, USA). Body temperature was measured rectally 3 to 5 days in row and mean values were calculated. Wt-mice were sacrificed during estrous phase of the cycle. atERαKO mice did not present a physiological estrous cycle, and were sacrificed independent of (non-) cycle phases; their vaginal smear was collected and analyzed to exclude a proestrous analogous stage. Organs were collected and frozen in liquid nitrogen. All animal experiments were approved by the Landesamt für Gesundheit und Soziales (LaGeSo, Berlin, Germany) and were conducted in alliance with the German Law on the Protection of Animals.

### Cell culture

THP-1 cells (American Type Culture Collection (ATCC)) were cultured in RPMI 1640 (Life Technologies) with 1% Pen-Strep (Invitrogen) and 10% charcoal-stripped fetal bovine serum (cs-FBS; Sigma Aldrich). THP-1 cells were differentiated for 48 hours with 10 ng/ml phorbol myristate acetate (Sigma Aldrich). Prior to stimulation, cells were kept in phenol red-free medium for 48 h and starved overnight (12 h) with phenol red-free RPMI medium reducing the content of cs-FBS to 2.5%. Next, cells were stimulated for 24 h with 10 nM E2 (Sigma), 50 μM solution of stearic acid (C18:0) in 10% BSA (Sigma Aldrich), or with 10% BSA alone (control). Lipopolysaccharide (100 nM, LPS) from *E. coli* (Sigma) was added 2 h prior to harvesting with lysis buffer (Qiagen). For luciferase reporter assay passive lysis buffer (Promega) was used.

Bone-marrow derived macrophages (BMDM) were isolated as described before^37^, using 10% L929-conditioned medium for macrophage differentiation. RPMI with 10% cs-FBS and 1% Pen-Strep was used to differentiate and cultivate cells. For stimulation, phenol red-free medium was used with addition of E2, BSA and C18:0 in the same concentrations as done with THP-1 cells.

HeLa cells (ATCC) were cultured in Dulbecco’s Modified Eagle Medium (DMEM) with addition of 10% cs-FBS and 1% Pen-Strep. Prior to stimulation with E2+BSA, or E2+C18:0 (analogously to THP-1 cells), cells were transfected as described below.

### Gene expression analysis

RNA from cultured THP-1 cells, and BMDM, from adipose tissue, uteri and hypothalamus was isolated with RNeasy Mini Kit (Qiagen) following the manufacturer’s instructions. For real-time PCR analysis, RNA was reverse transcribed and relative mRNA expression was normalized to 18 S (animal tissues), β2-microglobulin (BMDM) or to β-Actin (THP-1 cells). Primer sequences will be provided on demand.

### 17β-estradiol measurement in plasma

Plasma samples of atERαKO and wt mice were obtained after final blood collection. The measurement of 17β-estradiol (E2) concentrations was performed by a radioimmunoassay set up as a sequential assay as previously described^38^. Prior to radioimmunoassay, blood plasma was extracted twice with toluene and the pooled extracts were evaporated to dryness and re-dissolved. The antiserum used was directed against E2–6-carboximethyloxim (CMO)–BSA and exhibited the following cross-reactions: estrone, 1.3%; estriol, 0.7%; all tested non-phenolic steroids <0.01%. The minimum detectable concentration was 5 pg/ml; intra- and interassay coefficient of variation (CV) were 7.1 and 17.6%, respectively.

### Fatty acid profiling in plasma samples and in protein precipitates

Fatty acids were analysed in plasma samples from atERαKO mice on CD and HFD with a triplequad mass spectrometer coupled HLPC method, as described before^39^. Briefly, after hydrolysis, samples were diluted 1:10 in methanol containing internal standards. Samples were injected and separated by a reverse-phased column (Phenomenex Kinetex-C18 column 2.6 μm, 2.1 × 150 mm) with a solvent gradient containing aqueous formic acid (0.1%) and acetonitrile. After separation of components, fatty acid’s identity was analysed by an Agilent 6460 triplequad mass spectrometer with electrospray ionisation.

### Histology

After sacrificing mice, uteri were partly fixed with 4% formalin, and paraffin-embedded. Sections were deparaffinised with xylene and rehydrated with a gradient of alcoholic solutions. Antigen heat retrieval with citric acid and incubation with goat serum was performed with slides prior to hematoxylin and eosin (H&E), monoclonal rat anti-mouse Mac3 antibody (clone M3/84, BD Pharmingen, USA), and monoclonal rat anti-mouse Ly6G antibody (clone 1A8, Biolegend, USA) staining. For evaluation of the grade of inflammation, and for Mac3 and Ly6G expression a semi-quantitative scoring system was applied, using a standardized procedure under observation of the equivalent anatomical structures of the uteri.

### Flow cytometry

For FACS analysis, THP-1 macrophages were stained with PE conjugated anti-CD11b (BioLegend Inc. San Diego, USA) or PE conjugated anti-CD209 (BioLegend), and 7-AAD (Cell Viability Solution, BioLegend). FACS analysis was performed on a FACS Calibur flow cytometer (BD Biosciences, Heidelberg, Germany). As cells were only single stained, auto fluorescence controls were used to set negatives. The expression of the constitutively expressed adhesion protein CD11b and the M2-marker CD209 was quantified as mean fluorescence intensity (MFI). Relative up- or downregulation of CD11b- and CD209-expression was calculated by the following equation: MFI (CD11b (or CD209) of cells)/MFI (CD11b (or CD209) of E2+LPS treated cells). 7-AAD-staining was used to determine and exclude dead cells.

### Phagocytosis assay and immunofluorescence staining

For testing of phagocytosis activity, phagocytosis Assay Kit (Cayman) was used and performed in accordance to the manufacturer’s instructions. Briefly, FITC-labelled beads were added to the supernatant and THP-1 macrophages were processed analogously as for immunostaining experiments. For immunostaining cells were fixed with formalin and blocked with goat serum. Afterwards, cells were incubated with PE conjugated anti-CD209 antibody (Biolegend), washed, stained with DAPI (1:1000, Thermo Scientific, blue) and mounted with mounting medium (Dako). Three fluorescent pictures were captured randomly from every sample (n = 6 per group) with an all-in-one fluorescence microscope (BZ-9000E, Keyence). Finally, representative pictures were selected.

### Luciferase reporter assay

After plating and differentiating THP-1 cells as described above, cells were transiently transfected with hERα-pSG5 (kindly provided by P. Chambon, Institut Clinique de la Souris, Illkirch Cedex, France), or with pcDNA flag ERα wt or pcDNA flag ERα C447A (kindly provided by F. Acconcia, University Roma Tre, Rome, Italy), and with pERE-TkGL3 (kindly provided by P.J. Kushner, Metabolic Research Unit and Diabetes Center, University of California, San Francisco, USA), and pRL-CMV (Promega), a renilla luciferase vector. Transfection was performed with jetPEI^®^- Macrophage transfection reagent (Polyplus-transfection) according to the manufacturer’s instructions. Prior to stimulation, cells were starved for a maximum of 10 h. After harvesting, cell lysates were used to measure luciferase activity with the dual-luciferase reporter assay system (Promega).

### Immunoprecipitation

For ERα-immunoprecipitation, HeLa cells (ATTC) were transfected with 3 μg hERα-pSG5 or pSG5 using Lipofectamine^®^ 2000 (Thermo Scientific) and OptiMEM (Invitrogen). 24 h after transfection, cells were stimulated for 24 h with E2 and/or C18:0, in concentrations as described for THP-1 cells. Cells were lysed with RIPA (with proteinase-inhibitors, Complete Mini, Roche), sonicated (Sonopuls HD 2070, 30 s, 40–50%), and centrifuged. Supernatant was used to perform immunoprecipitation (IP) with anti-ERα-antibody (D8H8, Cell Signaling) bound to Protein A Sepharose-beads (Amersham). For western immunoblotting a distinct anti-ERα-antibody (sc-8002, Santa Cruz) was used. Washed beads-antibody-ERα complexes were analysed for fatty acids- binding as described above.

### Statistical analysis

All experiments were repeated at least three times and n-numbers are indicated for each experiment. Statistical analysis was performed with GraphPad Prism Software and statistical significance was assumed at p < 0.05. Comparison of mean values was evaluated by two-way ANOVA (Bonferroni posttest), two-way ANOVA with repeated measures (Bonferroni posttest), or unpaired t-tests, as appropriate.

## Additional Information

**How to cite this article**: Ban, Z. *et al*. High-Fat Diet Induces Unexpected Fatal Uterine Infections in Mice with aP2-Cre-mediated Deletion of Estrogen Receptor Alpha. *Sci. Rep.* **7**, 43269; doi: 10.1038/srep43269 (2017).

**Publisher's note:** Springer Nature remains neutral with regard to jurisdictional claims in published maps and institutional affiliations.

## Supplementary Material

Supplementary Information

## Figures and Tables

**Table 1:** Metabolic phenotyping of 6 weeks old female mice on CD.

	wt	atERαKO
Body weight [g]	19.28 ± 0.55	22.80 ± 0.41***
Locomotor acitivty [cnts/d]	91477 ± 10009	81981 ± 7452
Food intake [kcal/d]	12.50 ± 1.38	12.11 ± 0.85
Body temperature [°C]	37.36 ± 0.10	37.26 ± 0.11
Energy expenditure [kcal/h/kg lean mass]	27.93 ± 0.81	23.79 ± 0.69**
Lean mass [g]	15.30 ± 0.47	17.94 ± 0.64*
Fat mass[g]	1.96 ± 0.11	2.03 ± 0.06

N = 5. Two-tailed t-test. ^*^P < 0.05; ^**^P < 0.01; ^***^P < 0.001.
