# Supplementary material for: High-Fat Diet Induces Unexpected Fatal Uterine Infections in Mice with aP2-Cre-mediated Deletion of Estrogen Receptor Alpha
Source: Sci Rep. 2017 Feb 24;7:43269. doi: 10.1038/srep43269 (PMC5324142; doi:10.1038/srep43269)

High-Fat Diet Induces Unexpected Fatal Uterine Infections in Mice with aP2-Cre-mediated Deletion of Estrogen Receptor Alpha

Zsofia Ban[1], Paul Maurischat[1], Verena Benz[1], Sarah Brix[1], Anna Sonnenburg[2], Gerhard Schuler[3], Robert Klopfleisch[4], Michael Rothe[5], Jan-Åke Gustafsson[6], Anna Foryst-Ludwig[1,7,#], Ulrich Kintscher[1,7,#,*]

Supplemental data:

Fig. S1: Vaginal smears of a representative wt and atERαKO mice, obtained four days in a row

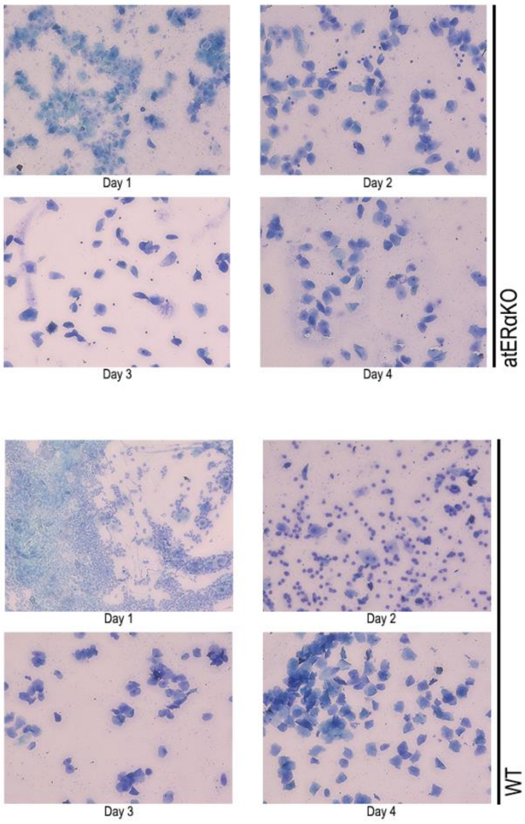

Fig. S2: Full-length Western Blot as control for IP (Inlay Fig. 5E)

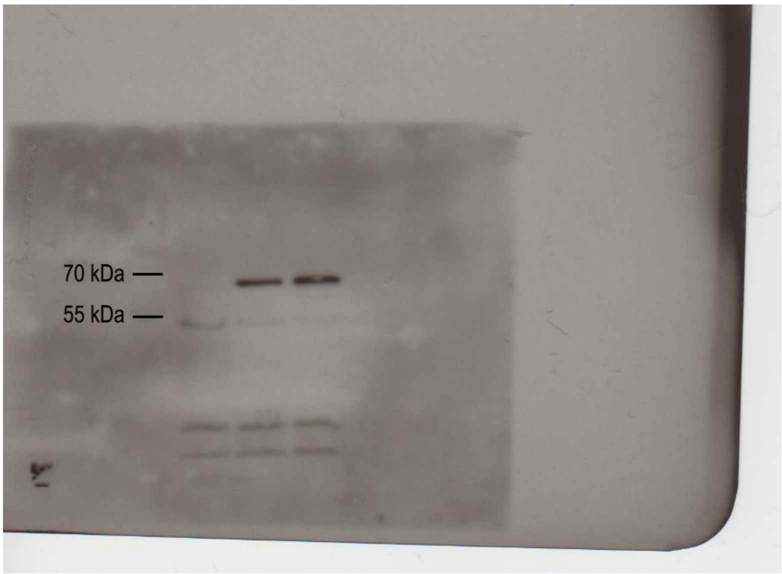

Supplement: Supplementary Information [file srep43269-s1.pdf]
